# A rapid and sensitive CRISPR-Cas12a for the detection of *Fusobacterium nucleatum*

**DOI:** 10.1128/spectrum.03629-23

**Published:** 2024-01-10

**Authors:** Hai Qu, Wenjing Zhang, Jianghao Li, Qingshan Fu, Xiaoxia Li, Miaomiao Wang, Guangyu Fu, Jing Cui

**Affiliations:** 1 Department of Pathogens, Medical College, Zhengzhou University, Zhengzhou, China; 2 Medical College, Henan University of Traditional Chinese Medicine, Zhengzhou, China; 3 Autobio Diagnostics Co., Ltd, Zhengzhou, China; Southern Medical University, Guangzhou, China

**Keywords:** *Fusobacterium nucleatum*, recombinase polymerase amplification, CRISPR-Cas12a, fluorescence-based detection, lateral flow immunoassay

## Abstract

*Fusobacterium nucleatum* (Fn), as a conditional pathogen, can cause a range of oral and gastrointestinal diseases. However, existing clinical detection methods require expensive equipment and complex procedures, which are inconvenient for large-scale screening in epidemiological research. The purpose of this study was to establish a reliable, rapid, and inexpensive detection method based on CRISPR/Cas12a technology for the detection of Fn. Specific recombinase polymerase amplification (RPA) primer sequences and crRNA sequences were designed based on the nusG gene of Fn. Subsequently, a fluorescence assay and a lateral flow immunoassay were established using the RPA and CRISPR-Cas12a system (RPA-CRISPR-Cas12a). Sensitivity validation revealed a limit of detection of 5 copies/µL. This method could distinguish Fn from other pathogens with excellent specificity. Furthermore, the RPA-CRISPR-Cas12a assay was highly consistent with the classical quantitative real-time PCR method when testing periodontal pocket samples. This makes it a promising method for the detection of Fn and has the potential to play an increasingly important role in infectious disease testing.

**IMPORTANCE:**

*Fusobacterium nucleatum* (Fn) naturally exists in the microbial communities of the oral and gastrointestinal tracts of healthy individuals and can cause inflammatory diseases in the oral and gastrointestinal tracts. Recent studies have shown that Fn is closely associated with the occurrence and development of gastrointestinal cancer. Therefore, the detection of Fn is very important. Unlike the existing clinical detection methods, this study established a fluorescence-based assay and lateral flow immunoassay based on the RPA and CRISPR-Cas12a system (RPA-CRISPR-Cas12a), which is fast, reliable, and inexpensive and can complete the detection within 30–40 minutes. This makes it a promising method for the detection of Fn and has the potential to play an increasingly important role in infectious disease testing.

## INTRODUCTION

*Fusobacterium nucleatum* (Fn) is a Gram-negative obligate anaerobic bacterium belonging to the *Fusobacterium* genus. It is a normal constituent of the microbiota of the oral cavity and gastrointestinal tract of healthy individuals. Fn has long been considered a conditional pathogenic bacterium that can cause inflammatory diseases in the oral cavity and gastrointestinal tract, such as periodontitis and gingivitis (1–3). Recent studies have shown that Fn is closely associated with the occurrence and progression of inflammatory bowel disease (4–10) and can cause appendicitis (11), and severe cases lead to the occurrence, development, and chemotherapy resistance of gastrointestinal cancer (12). The high occurrence of Fn in colorectal cancer tissues and feces may be a factor in the development of colorectal cancer (13, 14) and may be associated with chemoresistance to 5-fluorouracil in colorectal cancer (15). Furthermore, Fn is associated with various diseases not specific to the oral cavity and gastrointestinal tract, such as pericarditis (16), brain abscesses (17), and osteomyelitis (18).

The common methods for detecting Fn include bacterial isolation and identification, immunological detection, quantitative real-time PCR (qPCR), loop-mediated isothermal amplification (LAMP), and recombinase polymerase amplification (RPA). Traditional morphological identification methods require isolation and purification. Anaerobic bacteria are slow-growing and difficult to culture and often are accompanied by other bacteria in compound infections, making isolation and purification difficult; culture and identification time can take days or weeks to complete (19). These methods are not suitable for rapid detection and low-cost identification (20). An immunoassay method detects specific IgG and IgA in the serum of infected individuals, but its sensitivity and specificity are influenced by individual differences (21). qPCR requires a thermal cycler that runs for up to 90 min, so it is not suitable for rapid on-site detection. LAMP utilizes Bst DNA polymerase and multiple specific primers to amplify the target DNA sequence at around 60℃, but the primer design is complex and may result in false positives. RPA utilizes recombinase, polymerase, and specific primers to amplify the target DNA sequence at temperatures between 37°C and 42°C. RPA has a fast amplification speed, has high sensitivity, and only requires one pair of primers that is relatively simple to design, but a fluoroscope is needed after amplification. These methods have limitations in large-scale screening of infected patients, particularly in epidemiological studies. Therefore, there is an urgent need for rapid, accurate, simple, and portable techniques.

Fortunately, CRISPR technology has recently been used to detect infectious diseases (22–26). We established a CRISPR-Cas12a detection method for Fn based on the DNA Endonuclease-Targeted CRISPR *Trans* Reporter (DETECTR) to achieve rapid and accurate detection within 30–40 min. The entire testing process includes DNA extraction for 8–18 min, RPA amplification for 10 min, and CRISPR-Cas12a fluorescent detection for 10 min or CRISPR-Cas12a lateral flow immunoassay for 12 min.

## MATERIALS AND METHODS

### Bacterial strains

The tested strains were Fn and standard group strains of common gastrointestinal bacteria, as shown in Table 1. They were purchased from American Type Culture Collection (ATCC) and stored in Autobio Diagnostics Co., Ltd.

**TABLE 1:** Bacterial strains

Identification number	Bacteria name
ATCC25586	*Fusobacterium nucleatum*
ATCC25285	*Bacteroides fragilis*
ATCC19118	*Listeria monocytogenes*
ATCC13047	*Enterobacter cloacae* subsp. *cloacae*
ATCC12228	*Staphylococcus epidermidis*
ATCC13076	*Salmonella enterica*
ATCC49453	*Staphylococcus saprophyticus*
ATCC10031	*Klebsiella pneumoniae*
ATCC25923	*Staphylococcus aureus*
ATCC29212	*Enterococcus faecalis*
ATCC25922	*Escherichia coli*
ATCC27853	*Pseudomonas aeruginosa*
ATCC33693	*Fusobacterium periodonticum*

### Main reagents and instruments

The RPA amplification kit was purchased from TwistDX (Cambridge, UK). The Lba Cas12a enzyme was purchased from New England Biolabs (MA, USA). The primer, probe, and plasmid (containing the RPA-amplified fragment of Fn nusG gene) were obtained from GENERAL BIOL (Anhui, China). Nucleic acid extraction and purification were performed using reagents from TIANGEN (Beijing, China). Water purification was achieved with the Milli-Q system from Millipore (MA, USA). Real-time PCR was conducted using the 7500 instrument from Thermo Fisher Scientific (MA, USA). Streptavidin, sheep anti-mouse antibody, and anti-FITC mAb were obtained from Zhengzhou Immuno Biotech Co., Ltd. (Zhengzhou, China). The nitrocellulose membranes were purchased from Tianren Membrane of Science and Technology Co., Ltd. (Shaoxing, China). The conjugate pad, glass and cellulose fibers, absorption pad, and plastic adhesive board were obtained from Jiening Biotech Co., Ltd. (Shanghai, China). Lastly, the XYZ-HM3235 dispensing system, which enabled printing of lines with contact and dispensing of conjugate with non-contact, was provided by Kinbio Tech. Co., Ltd. (Shanghai, China).

### Bacterial culture and DNA isolation

The freeze-dried bacterial strain was added to 1.0 mL of sterile saline solution, mixed thoroughly, and used to inoculate a blood agar plate for activation. Incubation was carried out in an anaerobic environment (using an anaerobic bag or an anaerobic chamber) at 35–37℃ for 48 hours. A single colony from the cultured strain was diluted into the liquid culture medium; the bacteria were counted to ensure that the bacterial concentration was greater than 1 × 10^8^ CFU/mL. Bacterial genomic DNA was extracted using a TIANamp Micro DNA Kit (DP316, TIANGEN Biotech, Beijing, China) following the manufacturer’s protocol. Briefly, 200 µL of 1 × 10^6^ CFU/mL was used for extraction, and the DNA was eluted in 50 µL. Since the nusG gene is a single-copy gene, the copy number of Fn DNA was 4,000 copies/µL.

### Design of RPA primers and crRNAs

**Fig 1:**
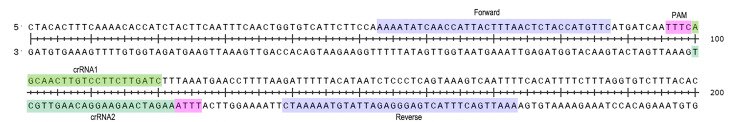
RPA primers and crRNAs design.

The National Center for Biotechnology Information (NCBI) database was used to analyze and compare the nusG sequence of Fn from which the RPA primers and crRNAs were designed (Fig. 1). The forward and reverse primers used in RPA were 5′-AAAATATCAACCATTACTTTAACTCTACCATGTTC-3′ and 5′-AAATTGACTTTACTGAGGGAGATTATGTAAAAATC-3′, respectively. There are two crRNAs: 5′-UAAUUUCUACUAAGUGUAGAUAGCAACUUGUCCUUCUUGAUC-3′ for crRNA1 and 5′-UAAUUUCUACUAAGUGUAGAUAAGAUCAAGAAGGACAAGUUGCU-3′ for crRNA2. RPA primers and crRNAs were synthesized by GENERAL BIOL (Anhui, China).

### Lateral flow biosensor preparation

Streptavidin (Zhengzhou Immuno Biotech Co., Ltd., Zhengzhou, China) and sheep anti-mouse antibody (Zhengzhou Immuno Biotech Co., Ltd., Zhengzhou, China) were dispensed onto an NC membrane (Tianren Membrane, Shaoxing, China) to form a control line and a test line with an XYZ-HM3235 dispensing system. The membrane was dried at 37℃ for 8 hours.

AuNPs with an average diameter of 40 nm were prepared according to Frens method (27). Briefly, 1 mL of 1% (w/v) trisodium citrate was added into a rapidly stirred and boiling 100 mL of 0.01% (w/v) HAuCl_4_ solution. The pH of AuNPs solution was adjusted to 8.0 with 0.1 M K_2_CO_3_. Then, 20 µg of anti-FITC antibody (Zhengzhou Immuno Biotech Co., Ltd., Zhengzhou, China) was added to 2 mL of AuNPs solution. After shaking at room temperature for 1 hour, 1% (w/v) BSA stock was added to the AuNPs solution, and 1 hour later, the resulting AuNPs solution was concentrated by centrifugation (10,000 rpm/4°C/20 min), the supernatant was removed, and the precipitate was resuspended with 0.5 mL of AuNPs solution (0.02 M PB pH 8.0 and 1% BSA). Finally, the AuNPs-binding anti-FITC antibody solution was dispensed onto a fiberglass conjugate pad.

The sample pad was prepared by soaking glass fiber in the sample pad buffer (0.02 M PB pH 8.0, 1% casein, and 0.5% Tween 20) for 1 hour. It was then dried for 8 hours at 37°C and stored in a low-humidity chamber at room temperature.

**Fig 2:**
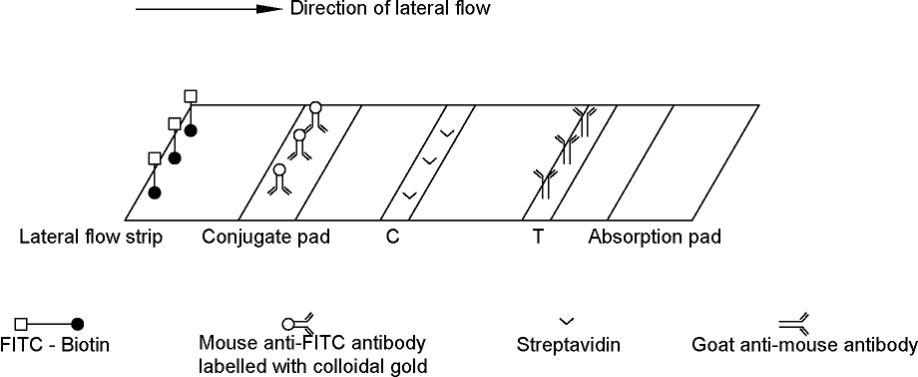
Composition of lateral flow immunoassay.

Sample pad, NC membrane, conjugate pad, and absorbent pad were attached along the PVC panels and cut into 3.0-mm strips. The test strips were assembled as shown in Fig. 2.

### RPA-CRISPR-Cas12a-Fn assay

The RPA reaction was run following the instructions of Twist Basic (TwistDx, Cambridge, UK). The reaction mix was incubated at 37°C for 5, 10, 15, 20, and 30 min, respectively, on an Applied Biosystems 7500 (Thermo Fisher Scientific, MA, USA).

RPA-CRISPR-Cas12a fluorescence assay is as follows: 2 µL of the RPA product was added to the CRISPR-Cas12a reaction system, an 18 µL mixture containing 13.4 µL DEPC-H_2_O, 2 µL NEBuffer 2.1, 0.4 µL of 5 µmol/L Lba Cas12a (Cpf1), 0.5 µL RNAse inhibitor (40 U/µL), 0.5 µL DTT (0.1 mmol/L) , 0.4 µL 0.01 mmol/L crRNA, and 0.8 µL of different concentrations of fluorescence probe (FAM-TTATT-BHQ1, 0.5, 1, 1.5, 2, and 2.5 µmol/L, respectively). The reaction mix was incubated at 37°C on the Applied Biosystems 7500 and real-time fluorescence curves were measured.

RPA-CRISPR-Cas12a lateral flow immunoassay is as follows: 2 µL of the RPA product was added to the CRISPR-Cas12a reaction system, an 18 µL mixture containing 12.2 µL DEPC-H_2_O, 2 µL NEBuffer 2.1, 0.4 µL 5 µmol/L Lba Cas12a (Cpf1), 0.5 µL RNAse Inhibitor (40 U/µL), 0.5 µL DTT (0.1 mmol/L), 0.4 µL 0.01 mmol/L crRNA, and 2 µL of different concentrations of lateral flow immunoassay probe (FITC-TTTTTTTTTT-Biotin, 0.5, 1, 1.5, 2, and 2.5 µmol/L, respectively). The reaction mixture was incubated at 37℃ for 10 min. For lateral flow immunoassay detection, the CRISPR-Cas12a reaction product was added to 80 µL ddH_2_O, and after mixing, a 50 µL mixture was loaded onto the sample pads, and the result was recorded 10 min later.

### Sensitivity of the RPA-CRISPR-Cas12a-Fn assay

To determine the detection limits of the RPA-CRISPR-Cas12a-Fn assay, a plasmid containing the RPA-amplified fragment of Fn nusG gene was diluted to 5 × 10^5^ copies/µL, 5 × 10^4^ copies/µL, 5 × 10^3^ copies/µL, 5 × 10^2^ copies/µL, 5 × 10^1^ copies/µL, 5 × 10^0^ copies/µL, and 1 copy/µL, respectively. Diluted plasmid (2 µL) was taken as the template at each concentration, and the lowest concentration detected by RPA-CRISPR-Cas12a fluorescent detection and RPA-CRISPR-Cas12a lateral flow immunoassay was used as the detection sensitivity.

### Specificity of the RPA-CRISPR-Cas12a-Fn assay

The bacterial strains in Table 1 were used for specificity assessment. A 200 µL of 1 × 10^6^ CFU/mL was used for extraction using a TIANamp Micro DNA Kit (DP316, TIANGEN Biotech, Beijing, China) following the manufacturer’s protocol. The extracts were tested by both RPA-CRISPR-Cas12a-Fn assay and lateral flow immunoassay. Fn was used as a positive control and water as blank control.

### Detection of clinical DNA sample by qPCR assay

According to the criteria for chronic periodontitis described by Caton et al. (28) and Tonetti et al. (29), we recruited 70 cases of periodontitis and collected periodontal specimens using the method described by Arenas et al. (30). The studies involving human participants were reviewed and approved. DNA extraction was performed using a TIANamp Micro DNA Kit. qPCR was performed according to the reported method of Arenas et al. (30).

### Statistical analysis

SPSS 19.0 software was used for statistical analysis. The results were expressed as means ± standard deviations of three independent experiments. Individual comparisons were made by chi-square test for paired data, and *P* values less than 0.05 were considered to be significant.

## RESULTS

### Establishment of RPA-CRISPR-Cas12a-Fn system

**Fig 3:**
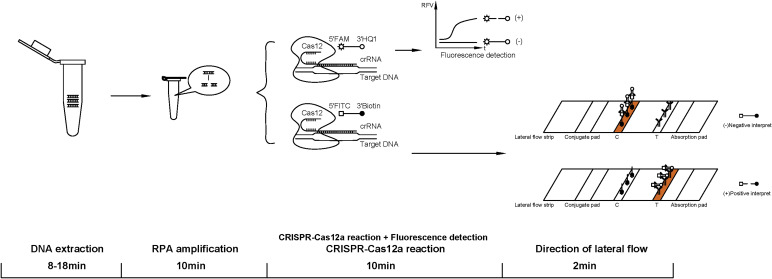
Schematic illustration of the RPA-CRISPR-Cas12a-Fn assay. After DNA extraction, the target sequence of Fn was amplified by RPA and then supplied to the CRISPR-Cas12a. The target sequence bonds with crRNA, activating the Cas12 to cleave the ssDNAs. The fluorescent-labeled system is detected using a fluorescent PCR instrument. The biotin-labeled system is monitored using a lateral flow strip.

By combining RPA with the CRISPR-Cas12a system, we developed a reliable and accurate detection method for the specific detection of Fn. This assay can be performed in a standard laboratory setting and provides rapid results within 30–40 min. DNA extraction using the direct lysis method can be completed within 8 min, while extraction using a kit requires 18 min. RPA amplification can be completed in 10 min. There are two signal detection methods: (i) using a fluorescent PCR instrument for CRISPR-Cas12a reaction and fluorescence detection, which can be completed in 10 min and (ii) using lateral flow immunoassay as the signal detection method, which requires 12 min, including a 10-min CRISPR-Cas12a reaction and a 2-min lateral flow immunoassay detection, as shown in Fig. 3.

**Fig 4:**
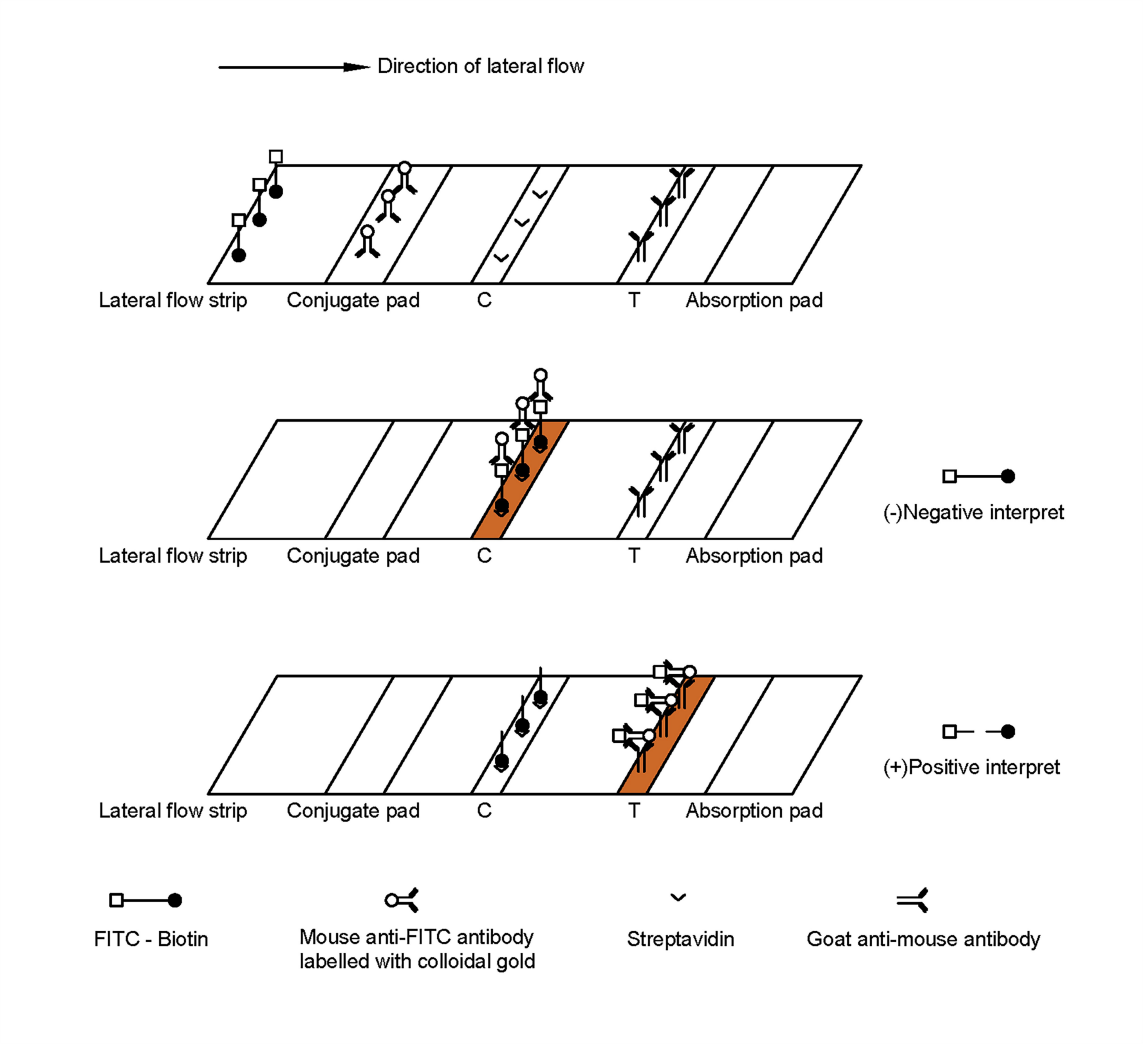
Schematic illustration of the RPA-CRISPR-Cas12a lateral flow immunoassay. Due to the absence of target DNA in negative samples, the biotin-FITC probe is not cleaved by Cas12a. It subsequently binds to AuNPs conjugated with anti-FITC antibodies and is captured by streptavidin at the control line (C-line), resulting in the appearance of a red band at the C-line. In positive samples containing target DNA, the biotin-FITC probe is completely cleaved. Biotin is captured by streptavidin at the C-line, while FITC, in combination with AuNPs conjugated with anti-FITC antibodies, cannot be captured at the C-line. Instead, it is immobilized at the test line (T-line) by goat anti-mouse antibodies, resulting in the appearance of a red band at the T-line.

To achieve rapid and convenient detection, we have developed a lateral flow immunoassay using a biotin-FITC probe (Fig. 4). This method allows for visual reading of the result, making it highly convenient and efficient.

### Selection of crRNA in RPA-CRISPR-Cas12a-Fn assay

**Fig 5:**
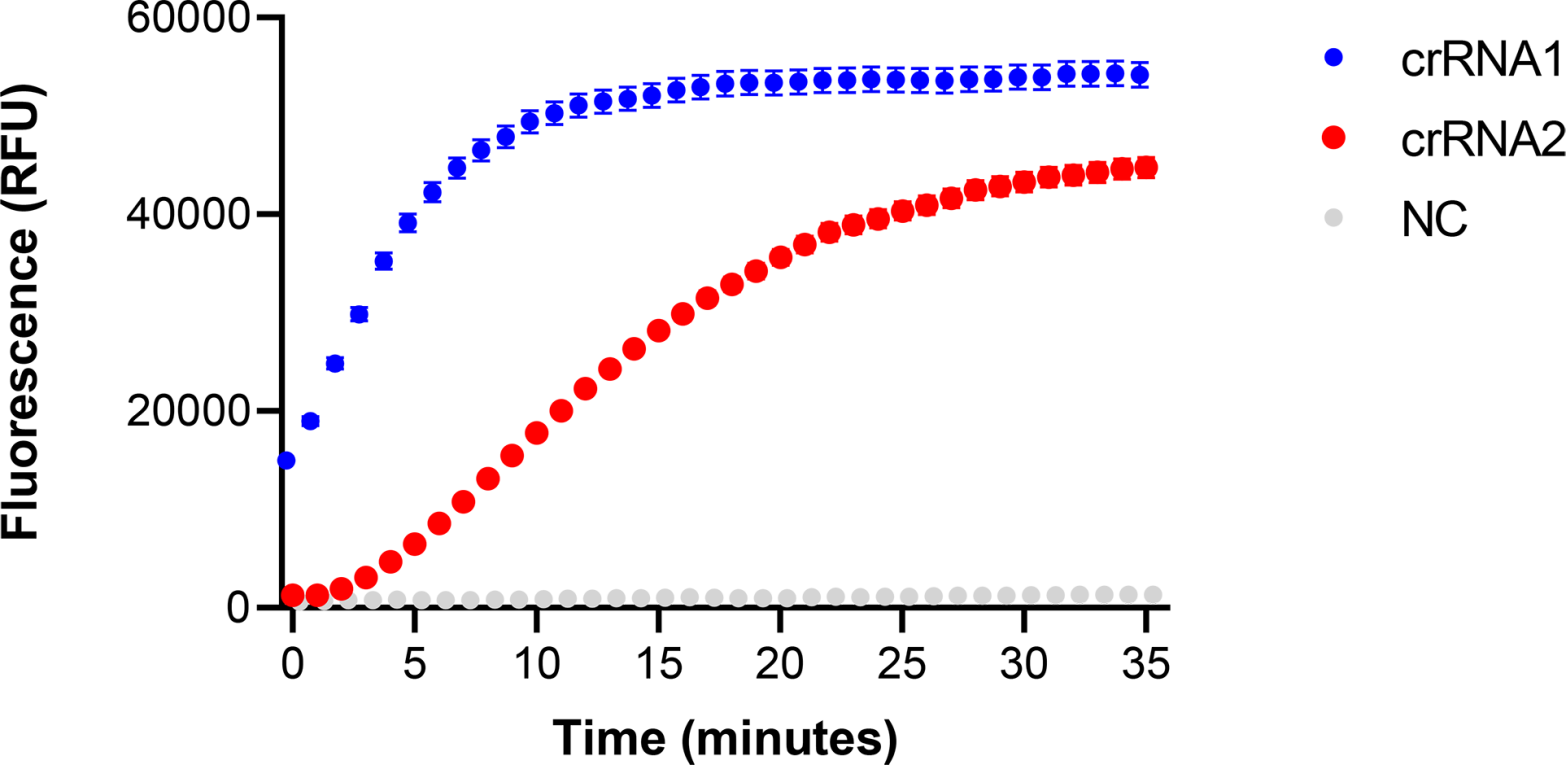
Real-time fluorescence signal of two crRNA designed for RPA-CRISPR-Cas12a-Fn assay; blue, crRNA1; red, crRNA2; and gray, without crRNA. Error bars represent ±SD, where *n* = 3 independent experiments.

The optimized crRNA for the CRISPR-Cas12a system was evaluated by fluorescence signal detection. To perform the fluorescence detection, the RPA was incubated at 37°C for 20 min and the CRISPR-Cas12a reaction system was incubated at 37°C for 35 min (35 cycles, 1 min each cycle). As shown in Fig. 5, the fluorescence signal of crRNA1 is higher than that of crRNA2. In addition, crRNA1 reached a plateau at 10 min, while crRNA2 continued to increase at 25 min. Therefore, we selected crRNA1 and a reaction time of 10 min for further investigation in the RPA-CRISPR-Cas12a-Fn assay.

### Optimization of RPA-CRISPR-Cas12a-Fn system

**Fig 6:**
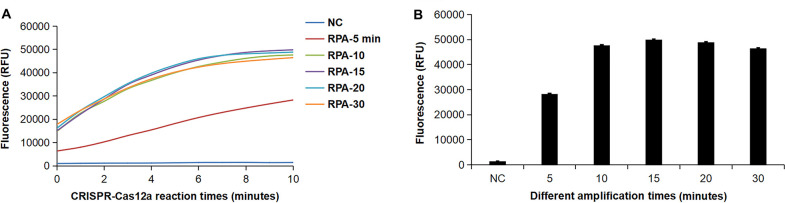
Optimized amplification time for the RPA reaction. (**A**) The fluorescence curves of RPA products generated with different amplification times. (**B**) The fluorescence intensity of RPA products with different amplification times is shown at 10 min. Error bars represent ±SD, where *n* = 3 independent experiments.

After determining the CRISPR-Cas12a reaction conditions, we optimized the amplification time for the RPA reaction. The reaction mix was incubated at 37°C for 5, 10, 15, 20, and 30 min. The fluorescence intensity of the RPA products generated at different amplification times was detected using the CRISPR-Cas12a reaction. As shown in Fig. 6A, there is no significant difference in fluorescence intensity after RPA amplification for more than 10 min. Furthermore, increasing the amplification time does not lead to a significant increase in fluorescence intensity (Fig. 6B). Therefore, we selected 10 min as the amplification time for the RPA reaction.

**Fig 7:**
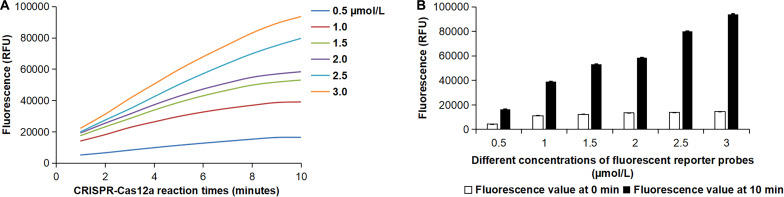
Optimized probe concentration in the RPA-CRISPR-Cas12a-Fn assay. (**A**) The fluorescence curves of different concentrations of the fluorescent reporter probe. (**B**) The fluorescence values of different concentrations of fluorescent reporter probes at 0 and 10 min during the CRISPR-Cas12a reaction. Error bars represent ±SD, where *n* = 3 independent experiments.

To ensure the accuracy of the detection results, we optimized the probe concentration in the RPA-CRISPR-Cas12a-Fn assay. The concentration of the fluorescent reporter probe (FAM-TTATT-BHQ1) was selected as 0.5, 1, 1.5, 2, 2.5, and 3 µmol/L. As shown in Fig. 7, the fluorescence intensity increases with the increase in the concentration of the fluorescent reporter probe. Additionally, the signal-to-noise ratio, which is the ratio of the fluorescence value at 10 min to the fluorescence value at 0 min, also improves. When the concentration of the fluorescent reporter probe is 1.5 µmol/L, the signal-to-noise ratio exceeds threefold, meeting the detection requirements. Therefore, we used a 1.5 µmol/L concentration of the fluorescent reporter probe in the RPA-CRISPR-Cas12a-Fn assay.

**Fig 8:**
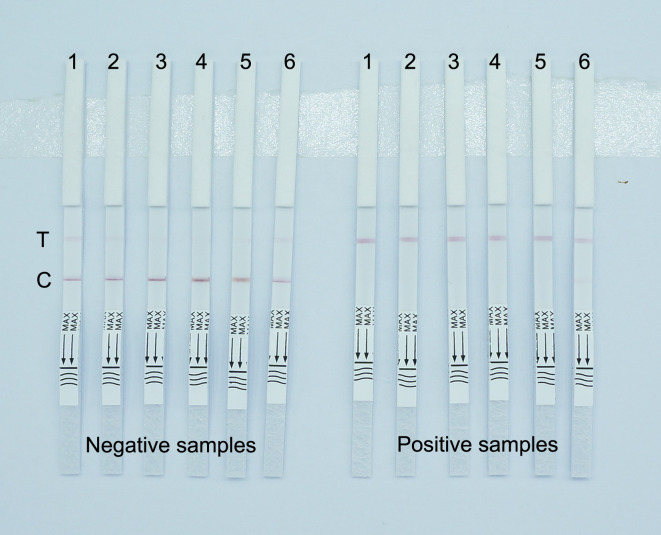
Images of RPA-CRISPR-Cas12a lateral flow immunoassay detection by different concentrations of the reporter probe. The fluorescence reporting probe concentrations represented by samples 1–6 are 0.5, 1, 1.5, 2, 2.5, and 3 µmol/L, respectively. C, control line; T, test line.

The concentration of the reporter probe in the RPA-CRISPR-Cas12a lateral flow immunoassay was set at 0.5, 1, 1.5, 2, 2.5, and 3 µmol/L for testing negative and positive samples (Fig. 8). During the detection of negative samples, when the probe concentration was less than 1.5 µmol/L, the AuNPs labeled with mouse anti-FITC antibodies could not be completely blocked by the C-line. On the other hand, when the probe concentration was too high, reaching 3 µmol/L, a HOOK effect occurred, resulting in faint red bands appearing at both the T-line and C-line. During the detection of positive samples, when the probe concentration was too high, up to 3.0 µmol/L, the probe may not have been completely cleaved during the CRISPR-Cas12a reaction, resulting in a faint red band appearing at the C-line. Therefore, both excessively high or low concentrations of the reporter probe lead to abnormal results. Taking into account the cost of detection, we determined that the optimal concentration of the reporter probe on the test strip should be 1.5 µmol/L.

### Sensitivity of the RPA-CRISPR-Cas12a-Fn assay

**Fig 9:**
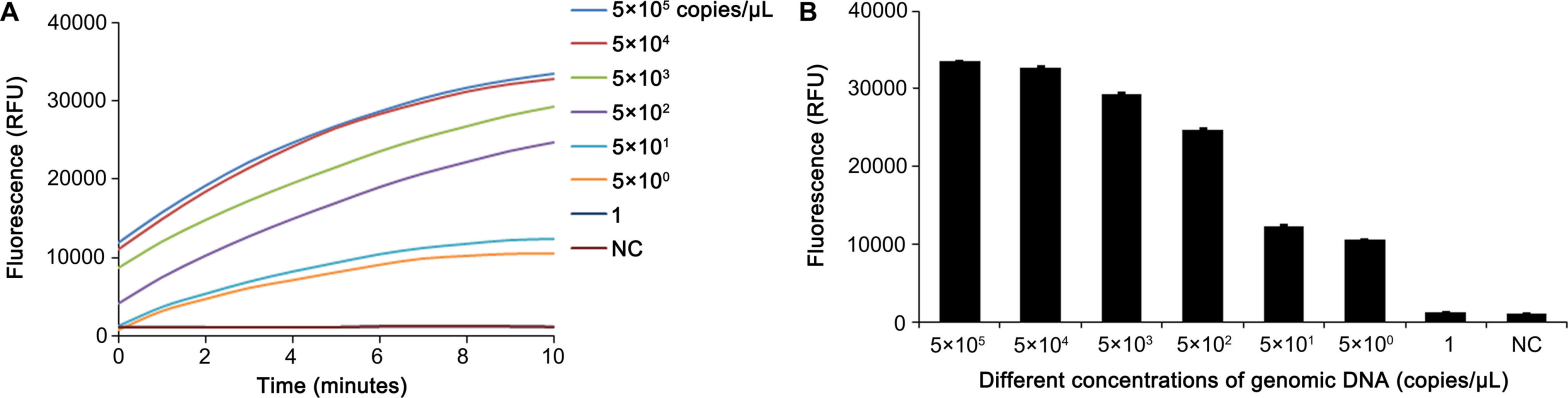
Sensitivity of the RPA-CRISPR-Cas12a-Fn fluorescent detection system. (**A**) The fluorescence curves of different concentrations of plasmid. (**B**) The fluorescence values of different concentrations of plasmid at 10 min during the CRISPR-Cas12a reaction. Error bars represent ±SD, where *n* = 3 independent experiments.

**Fig 10:**
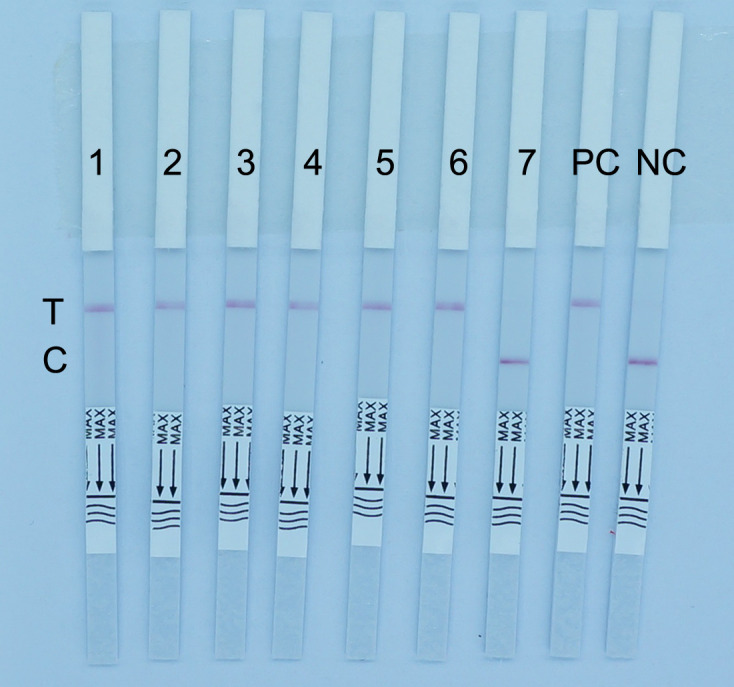
Images of RPA-CRISPR-Cas12a lateral flow immunoassay at different concentrations of plasmid. The concentrations of samples 1–9 are as follows: 5 × 10^5^ copies/µL, 5 × 10^4^ copies/µL, 5 × 10^3^ copies/µL, 5 × 10^2^ copies/µL, 5 × 10^1^ copies/µL, 5 × 10^0^ copies/µL, 1 copy/µL, PC, and NC. NC, non-template control; PC, Fn, C, control line; T, test line.

After the optimization of experimental conditions, the sensitivity of the RPA-CRISPR-Cas12a-Fn assay was evaluated using a serially diluted plasmid containing the RPA-amplified fragment of Fn nusG gene. The plasmid was diluted to 5 × 10^5^ copies/µL, 5 × 10^4^ copies/µL, 5 × 10^3^ copies/µL, 5 × 10^2^ copies/µL, 5 × 10^1^ copies/µL, 5 × 10^0^ copies/µL, and 1 copy/µL, respectively. Water was used as a blank control. The lowest detectable concentration by the RPA-CRISPR-Cas12a-Fn system was defined as the detection sensitivity. As shown in Fig. 9, the fluorescence assay of RPA-CRISPR-Cas12a revealed that the plasmid with a concentration of 5 copies/µL had a fluorescence value above 10,000, while the plasmid sample with a concentration of 1 copy/µL showed minimal fluorescence. Therefore, the sensitivity of the RPA-CRISPR-Cas12a-Fn fluorescent detection system was 5 copies/µL. The results of the RPA-CRISPR-Cas12a-Fn lateral flow immunoassay are shown in Fig. 10. A faint positive band was observed with a plasmid concentration of 5 copies/µL, while samples below 1 copy/µL did not show any significant positive bands. Hence, the limit of detection in the RPA-CRISPR-Cas12a-Fn lateral flow immunoassay is 5 copies/µL.

### Specificity of the RPA-CRISPR-Cas12a-Fn assay

**Fig 11:**
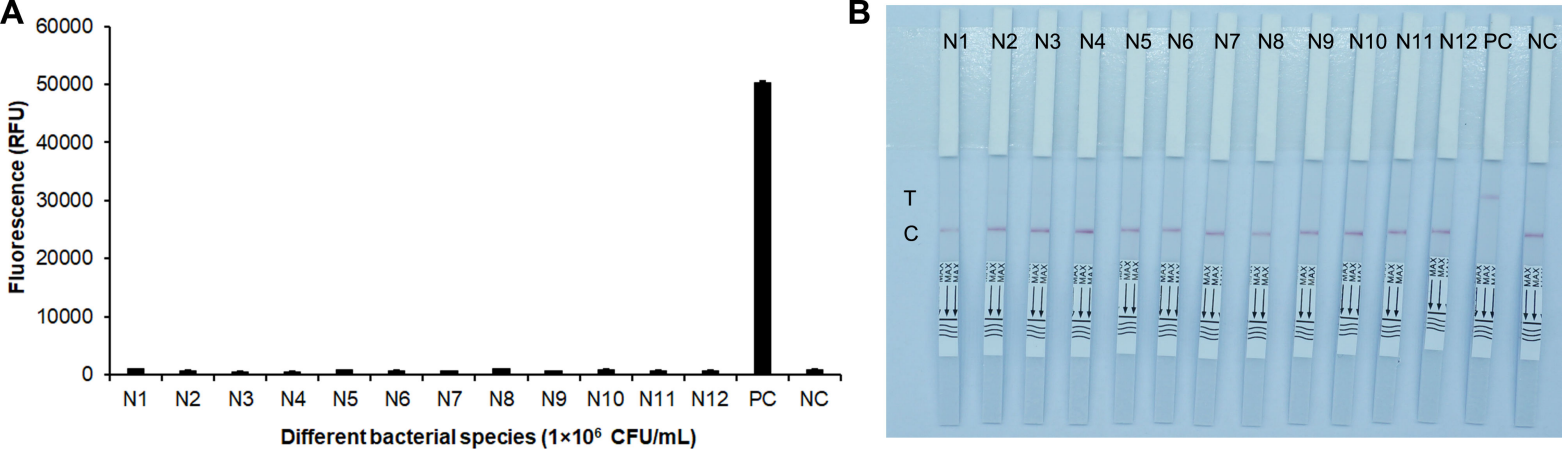
Specificity of RPA-CRISPR-Cas12a-Fn assays. (**A**) Results of RPA-CRISPR-Cas12a fluorescent assay. Error bars represent ±SD, where *n* = 3 independent experiments. (**B**) Results of RPA-CRISPR-Cas12a lateral flow immunoassay. NC, non-template control; PC, Fn; N1, *Bacteroides fragilis*; N2, *Listeria monocytogenes*; N3, *Enterobacter cloacae* subsp. *cloacae*; N4, *Staphylococcus epidermidis*; N5, *Salmonella enterica*; N6, *Staphylococcus saprophyticus*; N7, *Klebsiella pneumoniae*; N8, *Staphylococcus aureus*; N9, *Enterococcus faecalis*; N10, *Escherichia coli*; N11, *Pseudomonas aeruginosa*; N12, *Fusobacterium periodonticum*; C, control line; T, test line.

The bacterial strains in Table 1 were used for evaluating the specificity of the RPA-CRISPR-Cas12a-Fn assay. ddH_2_O was used for the negative control, and Fn was used for the positive control. As shown in Fig. 11, the RPA-CRISPR-Cas12a-Fn fluorescent assay showed that only Fn exhibited strong fluorescence signals, while other bacterial species did not show any significant fluorescence detection signals. The RPA-CRISPR-Cas12a lateral flow immunoassay showed distinct positive color bands specifically for Fn, while other bacterial species did not exhibit any positive color bands. Therefore, both methods demonstrate excellent detection specificity.

### RPA-CRISPR-Cas12a-Fn assay detection for periodontitis sample

**Fig 12:**
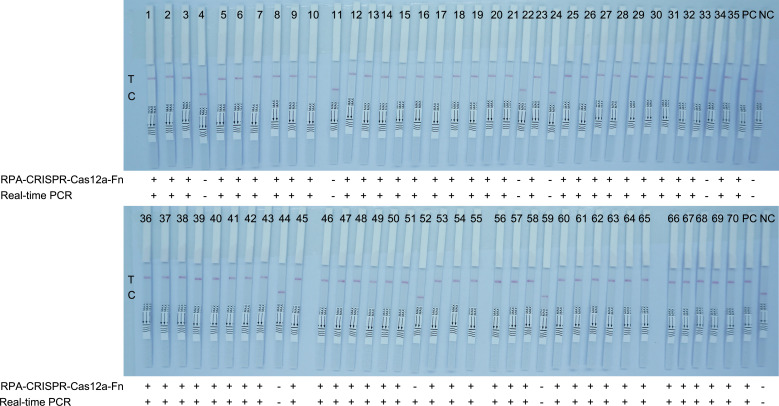
Results of 70 periodontitis patients’ periodontal pocket specimens analyzed by RPA-CRISPR-Cas12a-Fn lateral flow immunoassay and qPCR. NC, non-template control; PC, Fn; sample 1–70, clinical samples; C, control line; T, test line.

To evaluate the effectiveness of the RPA-CRISPR-Cas12a-Fn lateral flow immunoassay in complex samples and periodontitis patients, we tested the DNA samples from 70 periodontitis patients’ periodontal pockets and compared the results with the classical qPCR method. The study was approved by the Institutional Review Board (IRB) of the People’s Hospital of Zhengzhou, and the study conformed to the Declaration of Helsinki. The detection of RPA-CRISPR-Cas12a-Fn lateral flow immunoassay was completed within 40 min (Fig. 12). As shown in Table 2, there was no significant difference in the detection results between the two methods (*P* > 0.05), indicating that the RPA-CRISPR-Cas12a-Fn lateral flow immunoassay is reliable.

**TABLE 2:** Results of two detection methods in periodontitis population

Method		qPCR			Chi-square value	*P* value
		Positive	Negative	Total		
RPA-CRISPR-Cas12a-Fn assay	Positive	62	0	62	0.075	0.785
	Negative	1	7	8		
	Total	63	7	70		

## DISCUSSION

Looking back at our entire research process, we found that there are some key points that need further investigation in constructing a rapid and efficient RPA-CRISPR-Cas12a-Fn assay.

The lateral flow immunoassay is crucial for achieving convenient testing. During this study, we discovered that the preparation process of the lateral flow immunoassay may lead to false-positive results. Excessive AuNPs labeling of mouse anti-FITC antibodies can result in the complex of mouse anti-FITC antibody-labeled AuNPs/biotin-FITC probe that is not completely intercepted by the C-line. The excess complex continues to migrate and is captured by sheep anti-mouse antibodies fixed on the T-line, resulting in a red color appearing at the T-line. Additionally, if the streptavidin on the C-line is insufficient, it cannot completely intercept the complex. The excess complex continues to migrate and is captured by sheep anti-mouse antibodies fixed on the T-line, resulting in a red color appearing at the T-line position as well. Therefore, it is necessary to determine the appropriate amount of mouse anti-FITC antibody-labeled AuNPs and streptavidin for the assay.

During the establishment of the research system, two crRNAs were designed, which only differ by two nucleotides. However, significant differences were observed in the results during the study. CrRNA1 reached the reaction plateau rapidly within 10 min of the CRISPR-Cas12a reaction, and the fluorescence signal in the first 10 min was twice that of crRNA2. Therefore, the design of optimized crRNAs is a crucial factor in reducing the reaction time of the CRISPR-Cas12a reaction. In the optimization process of the RPA reaction conditions, the reaction reached the plateau within 10 min, indicating that the efficient reaction of RPA is an important factor for achieving rapid detection in RPA-CRISPR assays. Considering the reaction times of RPA and CRISPR-Cas12a, along with other operational times during the detection process, the entire RPA-CRISPR reaction can be completed within 30–40 min.

In terms of sensitivity and specificity, the RPA-CRISPR-Cas12a-Fn assay accurately detected 5 copies/µL. Furthermore, this method showed no cross-reactivity with *Fusobacterium periodonticum*, *Bacteroides fragilis*, *Listeria monocytogenes*, *Enterobacter cloacae* subsp. *cloacae*, and other pathogens, indicating excellent specificity. To evaluate the concordance rate of the RPA-CRISPR-Cas12a-Fn assay established in clinical sample detection, we conducted parallel comparisons with the qPCR method using clinical samples. The RPA-CRISPR-Cas12a-Fn assay yielded results highly consistent with the classical qPCR method. Moreover, the RPA-CRISPR-Cas12a-Fn assay had lower requirements for personnel, environment, and equipment, allowing for rapid sample testing and identification under specific conditions. In contrast, qPCR requires a more stringent operating environment, complex detection equipment, and higher demands on laboratory personnel, resulting in a longer overall testing time (31, 32). Therefore, the RPA-CRISPR-Cas12a-Fn assay has significant advantages over qPCR.

There are several limitations to this study. First, the sample types and sample size were limited, with only 70 samples included in our study, which may affect the conclusions regarding specificity and sensitivity. Second, although CRISPR technology offers advantages such as speed, accuracy, and convenience, the design of crRNA sequences is limited by the PAM sequence due to the requirement for specific recognition and activation of cutting activity by the Cas12a enzyme through the PAM sequence (33). As CRISPR technology continues to improve, it is hoped that our methods will play an increasingly important role in infectious disease testing.

In conclusion, we have established a rapid and sensitive method using CRISPR-Cas12a for the detection of Fn.

## SUPPLEMENTAL MATERIAL

Fig. S1 spectrum.03629-23-s0001.docx Results of 70 periodontitis patients’ periodontal pocket specimens analyzed by RPA-CRISPR-Cas12a-Fn lateral flow immunoassay and qPCR.

ASM does not own the copyrights to Supplemental Material that may be linked to, or accessed through, an article. The authors have granted ASM a non-exclusive, world-wide license to publish the Supplemental Material files. Please contact the corresponding author directly for reuse.
