# Supplementary material for: A rapid and sensitive CRISPR-Cas12a for the detection of Fusobacterium nucleatum
Source: Microbiol Spectr. 2024 Jan 10;12(2):e03629-23. doi: 10.1128/spectrum.03629-23 (PMC10845955; doi:10.1128/spectrum.03629-23)
Supplement: Fig. S1 — Results of 70 periodontitis patients’ periodontal pocket specimens analyzed by RPA-CRISPR-Cas12a-Fn lateral flow immunoassay and qPCR. [file spectrum.03629-23-s0001.docx]

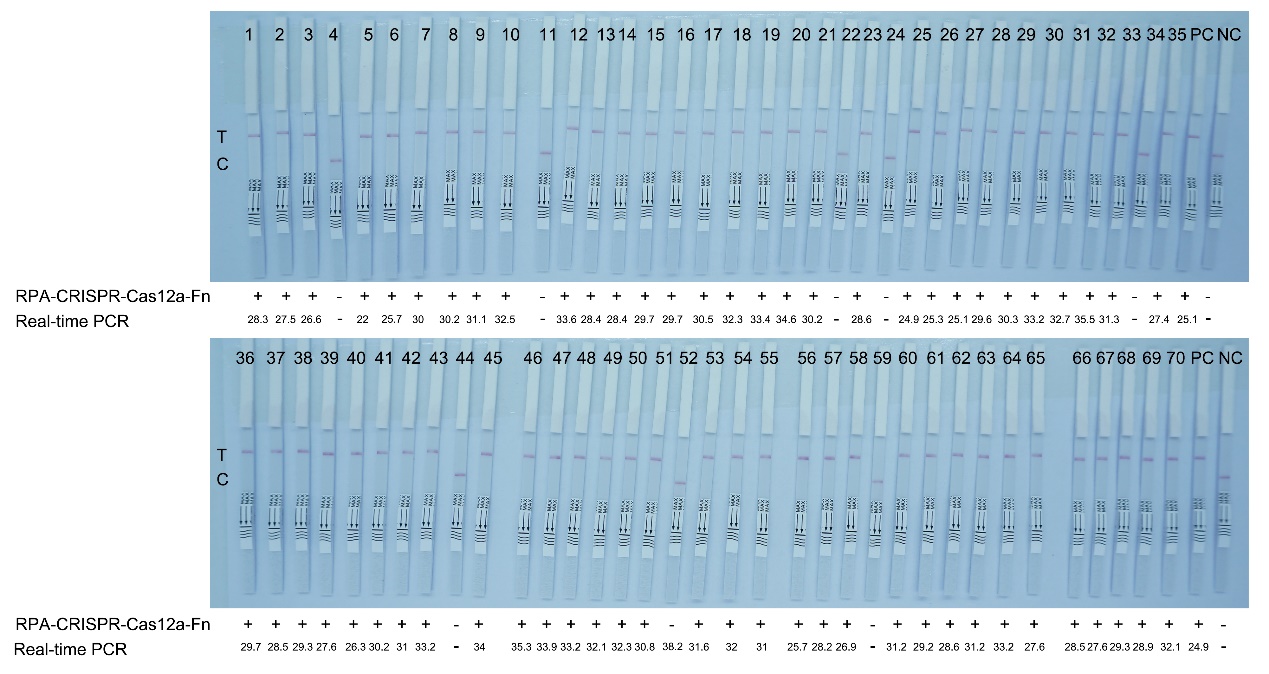


**FIG S1:** Results of 70 periodontitis patients' periodontal pocket specimens analyzed by RPA-CRISPR-Cas12a-Fn lateral flow immunoassay and qPCR. NC: non-template control; PC: Fn; Sample 1 ~ 70: clinical samples. C: Control line , T: Test line.
